# Prevalence and risk factors of diarrhea among children less than five years of age in the rural suburbs of Dire Dawa, Eastern Ethiopia; Robust Poisson Regression Analysis

**DOI:** 10.4314/ahs.v22i4.71

**Published:** 2022-12

**Authors:** Ephrem Tefera Solomon, Sirak Robele Gari, Bezatu Mengistie Alemu

**Affiliations:** 1 Haramaya University, College of Health and Medical Sciences, Harar, Ethiopia; 2 Ethiopian Institute of Water Resources, Addis Ababa University, Addis Ababa, Ethiopia

**Keywords:** Prevalence, diarrhea, rural Dire Dawa, Robust Poisson Regression

## Abstract

**Background:**

Diarrhea is the second leading cause of under-five mortality in Ethiopia. Information on the prevalence and the impacting factors of diarrhea in the rural suburbs of Dire Dawa is inadequate.

**Objective:**

This study was aimed at determining risk factors of diarrhea among children less than five years of age in the rural suburbs of Dire Dawa, eastern Ethiopia.

**Methods:**

A cross-sectional study was conducted from 02 May 2018, to 31 May 2018. The required 1146 children for this study were selected from the rural suburbs of Dire Dawa using multi-stage sampling. Both bi-variable and multivariable Robust Poisson regressions were used for identifying the risk factors. Explanatory variables with a p-value < 0.05 were considered as independently associated with diarrhea.

**Results:**

The prevalence of diarrhea among the children was 23% (95% CI: 20.7 - 25.4). The type of house floor (aPR [adjusted prevalence ratio] = 0.89, 95% CI: 0.84 - 0.95) and sharing latrine with neighbours (aPR = 1.18, 95% CI: 1.09 - 1.26) were the significant factors associated with childhood diarrhea.

**Conclusion:**

Earthen house floor and shared use of latrine were the risk factors associated with childhood diarrhea. Hence, improving the house floor condition and construction of private latrine together with health education are recommended.

## Introduction

Diarrhea is the second leading cause of death among children under the age of five^1^. In recent decades the world has made significant progress in reducing deaths from diarrheal diseases, especially among children less than five years of age, and mortality has fallen by two-thirds since 1990 2. In developing countries, an average of three episodes of diarrhea per child per year is reported^3^. Most of the deaths occur among children less than two years of age in South Asia and sub-Saharan Africa^4^. In Ethiopia, 23% of all under-five deaths – more than 70,000 children a year are due to diarrhea^5^. Thirty eight point four percent and 93% children less than five years of age in the country do not have access to improved water and improved sanitation, respectively 6. Although it has been known for decades that acute childhood diarrhea is the second leading cause of death for young children, the world is more aware of the contribution of gastroenteritis to the development of severe malnutrition, stunting, cognitive dysfunction and decreased adult accomplishment and productivity^7^.

The highest risk factors for development of diarrheal diseases are unsafe drinking water and poor sanitation^8^. Numerous studies distinguished the following risk factors: low socioeconomic status, faeces seen around the pit hole or on the floors of latrines, long distance from water source, living with animal in the same house, use of shared toilets, use of open bags for storing household waste, disposal of household waste on streets, improper waste disposal, unprotected water source and the housing floor material^9^–^16^.

In Ethiopia, childhood mortality has declined substantially since 2000. In 2016, the under-five mortality rate was 67 deaths per 1,000 live births. Under-five mortality in Dire Dawa was 93 deaths per 1,000 live births^17^. Nevertheless, the major causes of death for under-five children in Ethiopia are easily preventable and treatable. Of these, childhood diarrhea are the main cause of death for children 1 - 4 years followed by acute lower respiratory infection (ALRI) including pneumonia, severe acute malnutrition (SAM), measles, malaria, and meningitis^18^. The various studies conducted in the different vicinities of Ethiopia reported that the prevalence of childhood diarrhea ranged between 11% and 28.4% 10,11,13,16,19–24.

In order to keep the well-being of under-five children in line with the existing maternal and child health policy, it is crucial to pinpoint the impacting factors of diarrhea. The prevalence as well as the risk factors of diarrhea varies from place to place in Ethiopia. However, information on the prevalence and the risk factors of diarrhea in the rural suburbs of Dire Dawa is inadequate. Thus, the objective of this study was to assess the prevalence and identify factors associated with diarrheal disease among children less than five years of age in the rural suburbs of Dire Dawa.

## Methods

### Study area

Dire Dawa is one of the two city Administrations in Ethiopia. The Administration is situated in the eastern part of Ethiopia at about 515 km from Addis Ababa. The Dire Dawa Administration consists of Dire Dawa city and the surrounding rural areas. Rain fall pattern of the area is characterized by low rainy season from February to May; and high rainy season from July to September. The administration is located at an elevation ranging between 950 meters asl (above sea lvel) in the northeast to 2260 meters asl in southwest. The administration is divided into nine urban and 38 rural kebeles and the rural areas are served by seven health centers and 33 health posts. The Administration achieved a 100% access to primary healthcare in 2007^25^. However, in the rural area some people use water from protected sources such as springs, boreholes, deep and shallow protected wells, hand-dug wells. Others use from unprotected water sources such as surface water from river, open ditches, unprotected wells. As per the population projection for Ethiopia from 2007 - 2037, the population of Dire Dawa was projected to be 507,000 in 2020. Of these, 252,000 were males. More specifically, the rural population was projected to be 184,000 with 92,000 each for males and females^26^. Furthermore, the rural under-five children was projected to be 25, 927 in 2017; of which, 13,025 were males^26^.

### Study design and period

A community-based cross-sectional study was carried out in the rural suburbs of Dire Dawa from 02 May 2018 to 31 May 2018.

### Population

The source population is all mothers/caretakers with a minimum of one under-five child in the 38 rural suburbs of Dire Dawa. The study population is all mothers/caretakers with a minimum of one under-five child in the four randomly selected rural suburbs of Dire Dawa. Mothers/caretakers with under-five children who lived six and more months in the area were included. Mothers/caretakers who were critically ill and could not give response to questions as well as under-five children that had persistent diarrhea were excluded.

### Sample size determination

The required sample size for determining the prevalence of diarrhea was computed based on the single population proportion formula using the assumptions of 5% margin of error, 95% confidence level and 22.5% prevalence (the two week prevalence of diarrhea in children less than five years of age in Kersa Wereda, east Ethiopia)^27^. Accordingly, the required sample size was 268. However, the actual sample size was 1180 after taking into consideration the following requirements: 10% non-response rate and a design effect of four resulting from the use of multistage sampling for recruiting the study participants. A total of 1146 under-five children participated in this study with a response rate of 97.12%.

### Sampling procedure

The required sample for this study was selected from the rural suburbs of Dire Dawa using multi-stage sampling technique. First, all the 38 kebeles were listed alphabetically and simple random sampling technique (lottery method) without replacement was used to select four kebeles from the 38 rural kebeles. Second, simple random sampling technique without replacement was used again to select the study participants from “family folders” available from the Health Extension Workers' Offices. These folders are being updated regularly by the Health Extension Workers of the health centers and health posts. Probability proportional to size allocation was used to select the study households from the four kebeles. From eligible households with more than one under-five children, the study child was selected by simple random draw method.

### Data collection and quality assurance

The data collection tool was prepared using several documents such as published articles, EDHS (Ethiopia Demographic and Health Survey) and WHO (World Health Organization) core questionnaires associated with diarrhea. The data collection tool was first prepared in English and then translated to “Affan Oromo” which is the local language of the study area. To ensure the consistency of the translation the “Affan Oromo” version was translated back to English. Data were collected through face-to-face interview using the developed data collection tool. The questionnaire consists of questions categorized under socio-economic, WASH (Water, Sanitation and Hygiene) and child and environmental variables. The data was collected by 16 data collectors and four supervisors. The data collectors and supervisors were recruited from the local area based on the following criteria: “Affan Oromo” skills (for data collection) and high school graduation, (for supervision). Completeness and consistency of the questionnaires were checked daily by the supervisors. The data collectors and supervisors were in regularly frequent contact for the safe conduct of the study. The principal investigator trained the data collectors and supervisors on proper data collection methods. The training was held for two days and the data collection tool was pretested on the last day of the training in the surrounding kebele, which was not included in the actual study. The tool was, then, amended based on the findings of the pretest.

### Operational definition of terms

**Caretaker** is someone who looks after the under-five child in the absence of the biological mother

**Diarrhea** is the passage of three or more loose or liquid stools in a day^28^.

**Household head** is a person managing a household.

**Persistent diarrhea** is the abrupt onset of 3 or more loose stools per day persisting for more than 14 days^29^.

**Rural suburbs:** Rural kebeles surrounding the city of Dire Dawa.

### Statistical analysis

The generated data were edited, coded and entered into EPI-Data Version 3.1. Then, they were exported to SPSS version 23.0 for analysis. Descriptive statistics was used to summarize data. Modified (Robust) Poisson regression was used because its robust variance provides correct estimates and is a better alternative to the analysis of cross-sectional studies with binary outcomes than logistic regression, since the prevalence ratio is more interpretable and easier to communicate to non-specialists than the odds ratio 30. The bi-variable modified Poisson regression was used as a selecting criterion for those predictor variables to be incorporated in the multivariable modified Poisson regression based on their p-value scores. Only variables with a p-value < 0.1 at the bi-variable regression were included in the multivariable analysis of Poisson regression with robust standard errors to identify the exogenous factors of childhood diarrhea after adjusting for potential confounders. Hence, multivariable regression was performed to ascertain the risk factors of diarrhea among children younger than five years. Accordingly, four models were examined to identify the risk factors. Those explanatory variables with a p-values < 0.05 in the multivariable analysis were considered as independently associated with the outcome variable, diarrhea.

### Ethics

This research work was ethically reviewed and approved by the National Research Ethics Review Committee (NRERC) at Addis Ababa, Ethiopia. Official letter written by the Ethiopian Institute of Water Resources was given to Dire Dawa Regional Health Bureau; which consequently wrote a support letter to the respective health centers and health posts of the study area. Written consent was obtained from the mothers/caretakers of the under-five children after explaining to them the objective of the study as well as the merit and demerit of getting involved. The study participants' right, either not to participate or to withdraw was respected. Confidentiality of the information collected from the study households was maintained and the information was used only for this study. Mothers/caretakers were given advice to take their children with diarrhea to the closest health institution for better management of diarrhea.

## Results

### Prevalence of childhood diarrhea

In the present study, almost 50% of the households studied had one child under the age of five years. Majority, 1081 (94%) of mothers took their children with diarrhea to the nearby Health Post or Health Center. However, 34 (3%) of mothers gave oral rehydrating solution and 31 (2.7%) took no action. The mean birth weight of the children was 2970gm, (95% CI: 2930gm - 3009gm). Similarly, the mean daily household water consumption per capita per day was 15.48 L, (95% CI: 15.01 L - 15.92 L). About one-fourth of the children less than five years of age had diarrheal diseases (23%) (95% CI: 20.7 - 25.4).

### Socio-economic factors

Few households were led by mothers ([Unadjusted prevalence ratio] uPR = 0.99, 95% CI: 0.92 - 1.08). Three-fourth of the households was infested with flies (uPR = 1.02, 95% CI: 0.97 - 1.07). Most households had no: Radio (uPR = 1.03, 95% CI: 0.98 - 1.07), Television (uPR = 1.08, 95% CI: 1.01 - 1.15), Satellite receiving Dish (uPR = 1.17, 95% CI: 1.10 - 1.24), mobile phone (uPR = 1.04, 95% CI: 1.00 - 1.09), Bank saving account (uPR = 1.03, 95% CI: 0.96 - 1.10), and water storage tank (uPR = 0.91, 95% CI: 0.82 - 1.02). The presence of mobile phone, Television and Satellite receiving Dish in the households had a significant association with childhood diarrhea (Table 1).

**Table 1:** Childhood diarrhea associated with socio-economic factors in the suburbs of Dire Dawa, eastern Ethiopia, 2018

Factors	Childhood diarrhea		uPR⊙ with 95% (CI)◊

	No (%)	Yes (%)	
**Household head**			
Mother	58 (77.3)	17 (22.7)	0.99 (0.92 – 1.08)
Father	824 (76.9)	247 (23.1)	1
**Flies' infestation in the house**			
No	225 (78.7)	61 (21.3)	1
Yes	657 (76.4)	203 (23.6)	1.02 (0.97 – 1.07)
**Possess land**			
No	105 (77.2)	31 (22.8)	0.99 (0.94 – 1.06)
Yes	777 (76.9)	233 (23.1)	1
**Possess Television**			
No	803 (76.3)	250 (23.7)	1.08 (1.01 – 1.15)
Yes	79 (84.9)	14 (15.1)	1
**Possess Satellite receiving Dish**			
No	824 (76.0)	260 (24.0)	1.17 (1.10 – 1.24)
Yes	58 (93.5)	4 (6.5)	1
**Possess mobile phone**			
No	490 (74.7)	166 (25.3)	1.04 (1.00 – 1.09)
Yes	392 (80.0)	98 (20.0)	1
**Possess Radio**			
No	628 (76.1)	197 (23.9)	1.03 (0.98 – 1.07)
Yes	254 (79.1)	67 (20.9)	1
**Possess Bank saving account**			
No	794 (76.6)	242 (23.4)	1.03 (0.96 –1.10)
Yes	88 (80.0)	22 (20.0)	1
**Possess water storage tank**			
No	857 (77.3)	251 (22.7)	0.91 (0.82 – 1.02)
Yes	25 (65.8)	13 (34.2)	1

⊙ Unadjusted Prevalence Ratio

◊ Confidence Interval

### Water, Sanitation and Hygiene factors

Nearly half of the households get water on alternate days (uPR = 1.04, 95% CI: 0.99 - 1.09), the water storage container of one-fifth of the households were wide-mouthed (uPR = 1.09, 95% CI: 1.04 - 1.15) and a few numbers of households fetched water from the water source using uncovered container (uPR = 1.04, 95% CI: 0.95 - 1.14). It took more than half an hour for a round trip to fetch water for the two-fifth of the households (uPR = 1.05, 95% CI: 1.01 - 1.10) and in majority of the households the daily water consumption was less than 20L per capita per day (uPR = 1.01, 95% CI: 0.97 - 1.05). More than half of the households reported that they were throwing child's last stool in garbage (uPR = 0.98, 95% CI: 0.94 – 1.02). In majority of the households, soap was not available on the handwashing facilities (uPR = 1.02, 95% CI: 0.97 - 1.06) and most of the mothers/caregivers did not practice washing of the hands of their under-five children after defecation (uPR = 0.98, 95% CI: 0.93 - 1.04). Of these, the narrowness or wideness of the household's water storage container and the time taken to fetch water for a round trip had statistically significant association with childhood diarrhea (Table 2).

**Table 2:** Childhood diarrhea associated with WASH factors in the suburbs of Dire Dawa, eastern Ethiopia, 2018

Factors	Childhood diarrhea		uPR^⊙^ with 95% (CI)^◊^
	No (%)	No (%)	
**Water supply pattern from the water source**			
Every day	333 (80.0)	83 (20.0)	1
On alternate days	329 (75.6)	106 (24.4)	1.04 (0.99 – 1.09)
**The household water storage container**			
Narrow-mouthed	742 (78.9)	198 (21.1)	1
Wide-mouthed	140 (68.0)	66 (32.0)	1.09 (1.04 –1.15)
**Water bringing container from the water source**			
Covered container	840 (77.2)	248 (22.8)	1
Uncovered container	42 (72.4)	16 (27.6)	1.04 (0.95 – 1.14)
**Time taken to fetch water for a round trip**			
Less than half an hour	536 (79.5)	138 (20.5)	1
More than half an hour	346 (73.3)	126 (26.7)	1.05 (1.01 – 1.10)
**Daily water consumption per capita per day**			
Less than 20L	606 (76.7)	184 (23.3)	1.01 (0.97 – 1.05)
Greater than or equal to 20L	276 (77.5)	80 (22.5)	1
**Mother's knowledge about WaterGuard use**			
To make drinking water safe	770 (77.3)	226 (22.7)	1
I don't know	112 (74.7)	38 (25.3)	1.02 (0.96 – 1.08)
**Disposal of the child's last stool before the interview**			
Thrown in toilet	415 (75.7)	133 (24.3)	1
Thrown in garbage	467 (78.1)	131 (21.9)	0.98 (0.94 – 1.02)
**Presence of soap in the handwashing facility**			
No	683 (76.6)	209 (23.4)	1.02 (0.97 – 1.06)
Yes	199 (78.3)	55 (21.7)	1
**Washing the hands of under-five child after s/he defecated**			
No	612 (79.5)	158 (20.5)	0.98 (0.93 – 1.04)
Yes	149 (77.2)	44 (22.8)	1

### Child and environmental factors

Few children were not breastfed (uPR = 1.02, 95% CI: 0.97 - 1.08). One-fifth of the children had low weight at birth (uPR = 1.06, 95% CI: 1.00 - 1.10) and very few children had high weight at birth (uPR = 1.06, 95% CI: 0.95 - 1.18). Majority of the households had earthen floor (uPR = 1.15, 95% CI: 1.09 - 1.20). Some households used latrine occasionally (uPR = 1.04, 95% CI: 0.95 - 1.14) and few households shared latrine with their neighbours (uPR = 1.19, 95% CI: 1.10 - 1.28). In some households, faeces were seen surfaced around the pit hole and/or on the slab (uPR = 1.06, 95% CI: 1.02 - 1.11). The type of house floor, shared use of latrine with neighbours, and faeces seen around the pit hole and/or on the slab were significantly associated with childhood diarrhea (Table 3).

**Table 3:** Childhood diarrhea associated with child and environmental factors in the suburbs of Dire Dawa, eastern Ethiopia, 2018

Factors	Childhood diarrhea		uPR^⊙^ with 95% (CI)^◊^

	No (%)	No (%)	
**History of breastfeeding**			
No	155 (74.5)	53 (25.5)	1.02 (0.97 – 1.08)
Yes	727 (77.5)	211 (22.5)	1
**The child birth weight**			
Normal birth weight	684 (78.4)	188 (21.6)	1
Low birth weight	168 (72.7)	63 (27.3)	1.05 (1.00 – 1.10)
High birth weight	30 (71.4)	12 (28.6)	1.06 (0.95 – 1.18)
**The type of house floor**			
Earthen floor	745 (74.9)	250 (25.1)	1.15 (1.09 – 1.20)
Cement floor	137 (90.7)	14 (9.3)	1
**Frequency of latrine use**			
Sometimes	45 (75.0)	15 (25.0)	1.04 (0.95 – 1.14)
Always	315 (79.5)	81 (20.5)	1
**Sharing latrine with neighbors**			
No	287 (84.4)	53 (15.6)	1
Yes	73 (62.9)	43 (37.1)	1.19 (1.10 – 1.28)
**Feces seen around the pit hole and/or on the** **slab**			
No	573 (79.8)	145 (20.2)	1
Yes	309 (72.2)	119 (27.8)	1.06 (1.02 – 1.11)

### Diarrheal risk factors

A statistically significant difference in diarrheal cases was observed between those children who possessed Satellite receiving Dish than who did not possess Satellite receiving in the first model. Narrowness or wideness of the household water storage container and the time taken to fetch water for a round trip were significantly associated with diarrhea in the second model. The type of house floor and shared use of latrine had a significant relationship with diarrhea in the third model. Finally, in the fourth model two risk factors were independently associated with diarrhea i.e., the type of house floor and sharing latrine with neighbours.

Having controlled for all predictors of diarrheal disease, children living in households with cement floor were 0.11 times less likely to develop diarrhea than those children living in households with earthen floor ([adjusted prevalence ratio] aPR = 0.89, 95% CI: 0.84 - 0.95 and p < 0.001). In other words, children from earthen floor households were 1.12 times more likely to develop diarrhea than children from cement floor households. Similarly, after controlling for all predictors of diarrheal disease, those children living in households who shared latrine with neighbours were 1.18 times more likely to develop diarrhea than those children living in households who did not share latrine with neighbours (aPR = 1.18, 95% CI: 1.09 - 1.26 and p < 0.001) (Table 4).

**Table 4:** Risk factors analysis of diarrhea by multivariable modified Poisson regression among children less than five years of age in the suburbs of Dire Dawa, eastern Ethiopia, 2018

Risk factors	model I †aPR 95%( CI)	model II aPR 95%( CI)	model III aPR 95%( CI)	model IV aPR 95%( CI)
**Possess Television**				
No	0.98 (0.88 – 1.07)			0.99 (0.44 – 3.86)
Yes	1			1
**Possess Satellite receiving Dish**				
No	1.18 (1.06 – 1.30)‡			0.94 (0.85 – 1.04)
Yes	1			1
**Possess mobile Phone**				
No	1.03 (0.99 – 1.07)			0.98 (0.91 – 1.04)
Yes	1			1
The household water storage container				
Narrow-mouthed		0.92 (0.87 – 0.97).		0.97 (0.90 – 1.05)
Wide-mouthed		1		1
**Time taken to fetch water for a round trip**				
Less than half an hour		1		1
More than half an hour		1.05 (1.01 – 1.10)‡		1.06 (0.98 – 1.14)
**Type of house floor**				
Earthen floor			1	1
Cement floor			0.86 (0.82 – 0.91)‡	0.89 (0.84 – 0.95)‡
**Sharing latrine with neighbors**				
No			1	1
Yes			1.18 (1.10 – 1.27)‡	1.18 (1.09 – 1.26)‡
**Feces seen around the pit hole and/or on the** **slab**				
No			1	1
Yes			1.07 (0.99 – 1.15)	1.07 (0.99 – 1.15)

† Adjusted Prevalence Ratio

‡ *p* < 0.05

## Discussion

The present study determined the risk factors associated with diarrheal disease among children less than five years of age in the rural suburbs of Dire Dawa. The type of house floor and sharing latrine with neighbours were the identified independently associated risk factors.

The mean birth weight of the children in our study was 2.97 Kg, which is within the normal range of 2.50 Kg - 4.00 Kg^31^. Diarrheal morbidity is higher in children with low birth weight than with normal birth weight^32^. It has also been reported that children with a small size (low weight) at birth are more likely to have been associated with diarrheal morbidity than children who had a large size at birth 33. In our study, however, in the bivariate modified Poisson regression, neither low birth weight nor high birth weight had statistically significant effect on childhood diarrhea.

In this study, the mean daily water consumption per capita per day was 15.48 L, 95% CI: 15.01 - 15.92. This is actually less than the minimum daily required water of 20 L per capita per day within 1.5 Km distance. Nevertheless, the bivariate modified Poisson regression analysis showed the absence of association between daily water consumption and childhood diarrhea. In all, higher quantities of water in the home were generally associated with a lower odds of childhood diarrhea^34^.

The prevalence of diarrhea in our study was (23%) (95% CI: 20.7 - 25.4). This finding is comparable with studies carried out in Jamma district, South Wello zone, Northeast Ethiopia (23.1%) 13, in Benna Tsemay District, South Omo Zone (23.5%) 10, in Bahir Dar Zuria district (20%) 21, and in Hadaleala District Northeast Ethiopia (26.1%) 24. Nevertheless, our finding is lower when compared with studies conducted in Enderta Woreda Tigray Northern Ethiopia (35.6%) 35, and in Harena Buluk Woreda Oromia Region, South East Ethiopia (28.4%) 11 and higher when compared with studies carried out in Wolitta Soddo Town, Southern, Ethiopia (11%) 19, Dale District, Sidama zone, Southern Ethiopia (13.6%) 16, in Bahir Dar city, Northwest Ethiopia (14.5%)^23^, in Jigjiga town, Somali Regional State, eastern Ethiopia (14.6%)^22^, and in Debre Berhan town (16.4%)^20^. This could possibly be explained by differences in seasons of data collection, sample size and socio-cultural factors.

In the present study, the odds of diarrhea in children living in households with earthen floor were 1.12 times higher than children living in households with cement floor. This result is corroborated by findings reported from Dale District, Sidama Zone southern Ethiopia^16^, Ghana^36^ and Nigeria^14^. It is also in agreement with a concluding remark of a review on trends and risk factors for childhood diarrhea in sub-Saharan countries^33^. Children in households with floor material made from mud and sand were at a high risk of experiencing diarrheal episodes 14. This could probably be explained by the fact that children especially the younger ones crawling on the ground could swallow the diarrheal pathogens from contaminated earthen floor that is in contact with contaminated shoes from toilets. Unlike earthen floor, cement floor is easily cleanable to reduce the contamination rate. Our study found that the odds of diarrhea in children living in households sharing latrine with neighbours were 1.18 times higher than those who used unshared facilities. This is consistent with the results of studies carried out in Addis Ababa^37^, Kenya^38^, Ghana^15^ and Senegal^12^. It also agrees with the conclusion of a review by Fuller and colleagues (2014). In line with this, evidence from a review on shared sanitation facilities and the prevalence of diarrhea in young children obtained from 51 countries reported that sharing sanitation facilities appears to be a risk factor for diarrhea although differences in socioeconomic status are important. According to this review, in most countries, sharing appears to be harmful. However, in Nigeria and Cameroon, sharing appears to be protective, and in many other countries there was no difference in diarrhea prevalence attributable to sharing^39^. This might be due to inadequate attention given to shared toilets among neighbourhoods with regard to cleaning and maintaining the toilet, as well as constructing handwashing facilities nearby.

Although our study did not find a statistically significant association between childhood diarrhea and faeces-littered latrine there seems to be weak association as indicated by the p-value in the multivariable regression. Furthermore, similar surveys conducted elsewhere in Ethiopia: Nekemte town^40^, Addis Ababa^37^ and Benna Tsemay District, South Omo Zone^10^ proved the existence of these associations. Moreover, Well-equipped and more appropriately managed latrines could prevent child diarrhea more effectively than less equipped or inappropriately managed latrines^41^. On the contrary, if latrines are poorly managed, or used inappropriately, they can be potential disease transmission routes^42^. This is an indication that the cleanliness of a toilet is more important than the physical presence of the toilet itself in developing countries. The clearly observed faeces around the pit hole and or on the slab will create the favourable condition for flies to serve as vector for the transmission of the diarrheal pathogens to food and water that creates a vicious cycle of diarrhea.

Failure to consider seasonal variation in the occurrence of childhood diarrhea due to the limited time for data collection and direct data collection via face-to-face interview might have introduced reporting bias. However, we tried to prevent this through training the data collectors to encourage the mothers/caretakers of under-five children to report the truth only. Our incapacity of addressing all the potential risk factors of diarrhea is some of the limitations of this study.

## Conclusion

In summary, under-five diarrhea is still the unsolved health challenge of the community of rural Dire Dawa. Earthen floor of the house and shared uses of latrine with neighbors were the important risk factors identified in association with childhood diarrhea. In light of this finding, improving the house floor condition, construction of private latrine together with health education on how to maintain the cleanliness of earthen floors are recommended.

## Data Availability

The raw data used for writing this manuscript will be made available upon formal request from the principal investigator.

## Abbreviations

ALRI: Acute Lower Respiratory Infection
aPR: Adjusted Prevalence Ratio
ASL: Above Sea Level
CI: Confidence Interval
EDHS: Ethiopia Demographic and Health Survey
NRERC: National Research Ethics Review Committee
SAM: Severe Acute Malnutrition
uPR: Unadjusted Prevalence Ratio
WASH: Water, Sanitation and Hygiene
WHO: World Health Organization
